# Development and reproducibility evaluation of a Monte Carlo-based standard LINAC model for quality assurance of multi-institutional clinical trials

**DOI:** 10.1093/jrr/rru051

**Published:** 2014-06-23

**Authors:** Muhammad Nauman Usmani, Hideki Takegawa, Masaaki Takashina, Hodaka Numasaki, Masaki Suga, Yusuke Anetai, Keita Kurosu, Masahiko Koizumi, Teruki Teshima

**Affiliations:** 1 Department of Medical Physics and Engineering, Osaka University Graduate School of Medicine, 1-7 Yamadaoka, Suita, Osaka 565-0871, Japan; 2 Department of Radiology, Kaizuka City Hospital, 3-10-20 Hori, Kaizuka, Osaka 597-0015, Japan; 3 Department of Radiation Oncology, Osaka Medical Center for Cancer and Cardiovascular Diseases, 1-3-3 Nakamichi, Higashinari-ku, Osaka 537-8511, Japan; 4 Department of Accelerator Managing, Hyogo Ion Beam Medical Center, 1-2-1 Kouto, Shingu-cho, Tatsuno, Hyogo 679-5165, Japan

**Keywords:** Monte Carlo, quality assurance, LINAC model, commissioning, multi-institutional clinical trials, dose verification

## Abstract

Technical developments in radiotherapy (RT) have created a need for systematic quality assurance (QA) to ensure that clinical institutions deliver prescribed radiation doses consistent with the requirements of clinical protocols. For QA, an ideal dose verification system should be independent of the treatment-planning system (TPS). This paper describes the development and reproducibility evaluation of a Monte Carlo (MC)-based standard LINAC model as a preliminary requirement for independent verification of dose distributions. The BEAMnrc MC code is used for characterization of the 6-, 10- and 15-MV photon beams for a wide range of field sizes. The modeling of the LINAC head components is based on the specifications provided by the manufacturer. MC dose distributions are tuned to match Varian Golden Beam Data (GBD). For reproducibility evaluation, calculated beam data is compared with beam data measured at individual institutions. For all energies and field sizes, the MC and GBD agreed to within 1.0% for percentage depth doses (PDDs), 1.5% for beam profiles and 1.2% for total scatter factors (S_cp_s_._). Reproducibility evaluation showed that the maximum average local differences were 1.3% and 2.5% for PDDs and beam profiles, respectively. MC and institutions' mean S_cp_s agreed to within 2.0%. An MC-based standard LINAC model developed to independently verify dose distributions for QA of multi-institutional clinical trials and routine clinical practice has proven to be highly accurate and reproducible and can thus help ensure that prescribed doses delivered are consistent with the requirements of clinical protocols.

## INTRODUCTION

The planning and delivery procedures of radiotherapy (RT) have become increasingly complex with the introduction of three-dimensional (3D) treatment planning, and new challenges are continuously posed in response to the implementation of advanced technologies [1]. The concept of conformal dose distribution has also been extended to encompass both the physical and biological rationales for achieving the desired clinical results [2]. To optimize the composite dose distributions, intensity-modulated RT (IMRT) has been used to deliver non-uniform ﬂuence to the patient from any given position of the treatment beam [3]. While stereotactic body RT (SBRT) is used to deliver the extremely potent hypofractionated doses, several techniques, including small field targeting, motion management, compact dosimetry and image guidance, are used to achieve this task [4].

Advances in these technologies have resulted in the development of image-guided RT (IGRT), a procedure that uses image guidance at various stages. Specifically, IGRT has become a valuable tool for ensuring that the target is correctly located immediately before the dose is delivered [5].

Furthermore, the recent emergence of advanced technologies has drawn attention to the growing need for systematic quality assurance (QA) for routine clinical practice and multi-institutional clinical trials to enhance confidence about treatment quality and patient safety with available resources [6, 7]. The purpose of QA for multi-institutional clinical trials is to ensure that participating institutions deliver prescribed radiation doses that are clinically comparable and consistent with the requirements of clinical trial protocols, resulting in a more robust validation of the outcomes of these trials [8, 9].

The Advanced Technology Consortium (ATC) has pioneered support for QA methods and data management for multi-institutional clinical RT trials. For trials related to IMRT and/or SBRT, each participating institution must pass a credentialing process. As part of this process, each institution must successfully irradiate a standardized phantom and is required to complete a protocol-specific Dry-Run test [10]. Therefore, QA procedure for participation assures consistency between calculated and delivered dose distributions in a homogeneous or inhomogeneous phantom. Although, after credentialing, submitted treatment plans are reviewed in terms of contouring of targets and organs at risk and their corresponding dose–volume histograms, it is necessary to independently verify the calculated dose distributions in a patient. As a general requirement, an ideal dose verification system should be independent of the treatment-planning system (TPS). Monte Carlo (MC) methods have played an important role in general TPS IMRT QA and patient-specific IMRT QA [11–12]. For multi-institutional clinical trials and routine clinical practice, the MC algorithm is most suitable for the verification of dose distributions in a patient because it is the most accurate algorithm available for independent dose calculations that can account for tissue inhomogeneity in a patient and dosimetric effects generated by multi-leaf collimators (MLCs) [13–15]. Although it would be best to create models of individual LINACs used at participating institutions, this would be a very time-consuming process and thus impractical. Instead, development of a vendor-specific standard LINAC model with standard beam data would be more efficient because only one MC commissioning process would be sufficient and thus efficacious for verification of submitted dose distributions.

The purpose of the study reported here was to develop an MC-based standard LINAC model as a preliminary requirement to independently verify dose distributions submitted by participating institutions for the QA of multi-institutional clinical trials and routine clinical practice. We developed MC beam models for three Varian photon beams (6, 10 and 15 MV) and a wide range of field sizes (from 3 × 3 to 40 × 40 cm^2^). MC dose distributions were tuned to match Varian golden beam data (GBD) very precisely. For reproducibility evaluation, calculated beam data was compared with beam data measured at individual institutions. Percentage depth doses (PDDs), beam profiles, total scatter factors (S_cp_s) and doses at calibration point (i.e. depth of 10 cm) under calibration conditions were collected. To perform statistical analysis, the means and standard deviations of average local differences were calculated, and the 95% confidence level was used to estimate the probability range.

## MATERIALS AND METHODS

### Monte Carlo modeling of standard LINAC

Our LINAC model is based on the Monte Carlo Verification System (MCVS). The MCVS is an integrated infrastructure developed for the verification of the accuracy of conventional dose distributions [16]. It consists of a graphical user interface for performing MC simulations and analyzing dose distributions. The dose calculation engine has been developed using BEAMnrc and DOSXYZnrc user codes [17–18]. BEAMnrc simulations of the treatment head of the linear accelerator are divided into two parts, i.e. the patient-independent part and patient-dependent part. The modeling of the geometry and composition of the LINAC head components (target, primary collimator, flattening filter, monitor chamber and secondary collimator jaws) are based on the specifications provided by the manufacturer.

Originally the MCVS was developed to commission a 6-MV photon beam from the Varian CLINAC 23EX with the Millennium MLC (Varian Medical Systems, Palo Alto, CA, USA) installed at Osaka Medical Center for Cancer and Cardiovascular Diseases. The MC results agreed with the ionization chamber measurements to within 1.0%.

The LINAC model developed with the MCVS can be used as a basis for a standard LINAC except for target and flattening filter because the components of the model are based on a Varian high-energy accelerator series.

The MCVS was originally developed for 6-MV photon beams only. To increase the range of applicable energies, new models for the targets and flattening filters of 10- and 15-MV photon beams were created and introduced into the MCVS based on the specifications provided by the manufacturer.

### Beam commissioning with Golden Beam Data

Parameters of incident electron beams, such as the mean energy, radial intensity distribution and divergence, were adjusted by comparing them with Varian GBD. The GBD represents the beam characteristics (PDDs, beam profiles and output factors) with a high degree of excellence provided by the manufacturer for use as a reference for the commissioning of Varian linear accelerators. The validation of the development process follows a fine-tuning procedure for adjustment of the parameters. A large number of iterations for adjustment of the electron beam parameters was performed until agreements between the MC and GBD PDDs and dose profiles were within the predefined acceptance criteria [14, 19–20].

PDDs along the beam central axis, dose profiles and S_cp_s for three photon beams (6-, 10- and 15-MV) for a wide range of field sizes (3 × 3 cm^2^, 10 × 10 cm^2^, 20 × 20 cm^2^, 30 × 30 cm^2^ and 40 × 40 cm^2^) were calculated and compared with those of GBD.

Overall phantom and voxel dimensions (*X*, *Y*, *Z*, where *Z* is depth) were set at 50 × 50 × 50 cm^3^ and 0.25 × 0.25 × 0.50 cm^3^, respectively. A water phantom was used as the ‘patient geometry’ for all simulations. With the gantry assumed to be at 0^o^ for all simulations, the phantom was placed at a source-to-surface distance (SSD) of 100 cm for calculations of PDDs and beam profiles. The beam profiles were calculated at depths of 5, 10, 20 and 30 cm. For each point, the local difference relative to GBD at that point was calculated. For S_cp_ calculations, phantom and voxel dimensions were set at 50 × 50 × 50 cm^3^ and 0.25 × 0.25 × 0.50 cm^3^, respectively. The calculations were performed with a SSD of 95 cm and a depth of 5 cm.

### Reproducibility evaluation of the standard LINAC model

To evaluate the reproducibility of the standard LINAC model thus developed, calculated beam data was compared with beam data measured at individual institutions. A total of 16 sets of measured beam data were provided from seven institutions for the following LINACs: five Clinac iX, two Clinac 2100CD, and one Clinac 23EX. Of these 16 beam datasets, four were for 4-MV, four for 6-MV, seven for 10-MV, and one for 20-MV photon beams.

We collected the data for PDDs, beam profiles, S_cp_ and doses at the calibration point (i.e. a depth of 10 cm) under calibration conditions.

All beam data were measured in accordance with professional guidelines, such as those specified in AAPM Task Group Reports Nos 45 and 106 [21–22]. The PDDs and beam profiles were measured with an SSD of 100 cm and S_cp_ with an SSD of 90 cm and a depth of 10 cm. According to field size, appropriate chambers were selected to measure these data at each institution.

For PDDs and beam profiles, the mean differences and standard deviations were calculated for low dose gradient areas only (i.e. the points in the build-up and penumbra region were excluded).

### Details of the Monte Carlo dose calculations

MC calculations were performed on the cluster at the Cyber Media Center, Osaka University, which consists of 128 nodes with four CPUs per node. Particle production and transport thresholds for all simulations were ECUT = 0.7 MeV and PCUT = 0.01 MeV. Range rejection was not employed.

Since it has been demonstrated [23] that the use of photon splitting with phase space data can sufficiently enhance the efficiency of dose calculations in simulated photon beams, photon splitting was employed in our study. Phase space data were not restarted at any time, as this leads to an underestimation of the statistical uncertainty. In our study, the statistical uncertainty for the total dose was generally <1.0%. The differences between MC and GBD dose distributions were normalized to the local dose rather than the maximum dose.

## RESULTS

### 6-MV photon beams

**Fig. 1.**
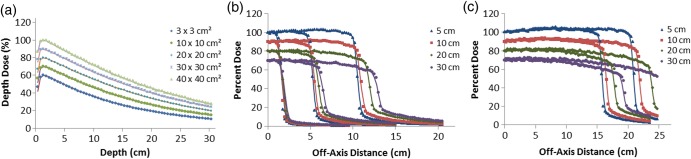
Comparison between MC (symbols) and GBD (lines): (**a**) PDD curves and (**b**), (**c**) dose profiles for 6-MV photon beam for 3 × 3 cm^2^, 10 × 10 cm^2^, 20 × 20 cm^2^, 30 × 30 cm^2^ and 40 × 40 cm^2^ fields. For clarity, the 30 × 30 cm^2^, 20 × 20 cm^2^, 10 × 10 cm^2^ and 3 × 3 cm^2^ fields are scaled by 0.9, 0.8, 0.7 and 0.6 respectively for PDD curves. Beam profiles are calculated at the depths of 5, 10, 20 and 30 cm. Curves for the depths of 10, 20 and 30 cm are scaled by 0.9, 0.8 and 0.7, respectively, for inclusion on the same graph. All curves are normalized to dose at d_max_ of central axis. Calculations are based on optimal parameters.

PDD curves for the 6-MV beam for the 3 × 3 cm^2^, 10 × 10 cm^2^, 20 × 20 cm^2^, 30 × 30 cm^2^ and 40 × 40 cm^2^ fields, obtained with optimal primary electron beam parameters, are shown in Fig. 1a, which demonstrates good agreement between MC and GBD: for all doses below d_max_, the average local difference between the MC and GBD PDD curves was <0.7%. Figure 1b and c show the 6-MV dose profiles for the 3 × 3 cm^2^, 10 × 10 cm^2^ and 20 × 20 cm^2^ and for the 30 × 30 cm^2^ and 40 × 40 cm^2^ fields, respectively.

For each field size, four different dose profiles were acquired at the depths of 5, 10, 20 and 30 cm and the average local difference between the MC and GBD for all fields in the plateau region (the central region of the beam profile bounded by 80% of the field width) was within 1.5%. In the penumbra region (the lateral distance between the 20% and 80% dose level at the depth of D_max_), a maximum difference of <1.5 mm distance to agreement (DTA) was attained for all curves, while the MC and GBD S_cp_s agreed to within 1.0% (Table 1).

**Table 1.** Comparison between MC and GBD S_cp_s for 6-, 10- and 15-MV photon beams: the MC S_cp_s are based on the optimal primary electron parameters

Field size (cm)	MC S_cp_	GBD S_cp_	% Diff
**6 MV**			
3 × 3	0.887	0.879	0.9
10 × 10	1	1	
20 × 20	1.061	1.066	−0.5
30 × 30	1.098	1.102	−0.4
40 × 40	1.114	1.126	−1.0
**10 MV**			
3 × 3	0.892	0.884	0.9
10 × 10	1	1	
20 × 20	1.051	1.058	−0.7
30 × 30	1.085	1.089	−0.4
40 × 40	1.096	1.110	−1.2
**15 MV**			
3 × 3	0.898	0.893	0.6
10 × 10	1	1	
20 × 20	1.038	1.045	−0.7
30 × 30	1.059	1.069	−0.9
40 × 40	1.077	1.085	−0.7

### 10-MV photon beams

**Fig. 2.**
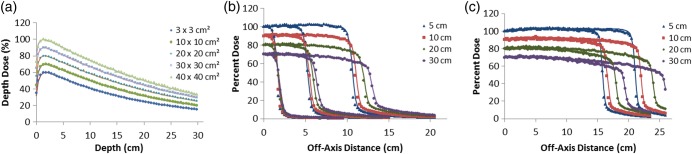
Comparison between MC (symbols) and GBD (lines): (**a**) PDD curves and (**b**), (**c**) dose profiles for 10-MV photon beam for 3 × 3 cm^2^, 10 × 10 cm^2^, 20 × 20 cm^2^, 30 × 30 cm^2^ and 40 × 40 cm^2^ fields. For clarity, the 30 × 30 cm^2^, 20 × 20 cm^2^, 10 × 10 cm^2^ and 3 × 3 cm^2^ fields are scaled by 0.9, 0.8, 0.7 and 0.6 respectively for PDD curves. Beam profiles are calculated at the depths of 5, 10, 20 and 30 cm. Curves for the depths of 10, 20 and 30 cm are scaled by 0.9, 0.8 and 0.7, respectively, for inclusion on the same graph. All curves are normalized to dose at d_max_ of central axis. Calculations are based on optimal parameters.

The 10-MV PDD curves for all field sizes are shown in Fig. 2a. Dose distributions for MC agreed with those for GBD to within 0.5% of average local differences for all doses below d_max_. Figure 2b and c show the 10-MV dose profiles for the 3 × 3 cm^2^, 10 × 10 cm^2^ and 20 × 20 cm^2^ and for the 30 × 30 cm^2^ and 40 × 40 cm^2^ fields, respectively. There was good agreement between the MC and GBD dose profiles: for all curves the average local difference between the MC and GBD in the plateau region was within 1.0%. For the penumbra region, the agreement was generally within 1 mm DTA for all fields, while the MC and GBD S_cp_s agreed to within 1.2% (Table 1).

### 15-MV photon beams

**Fig. 3.**
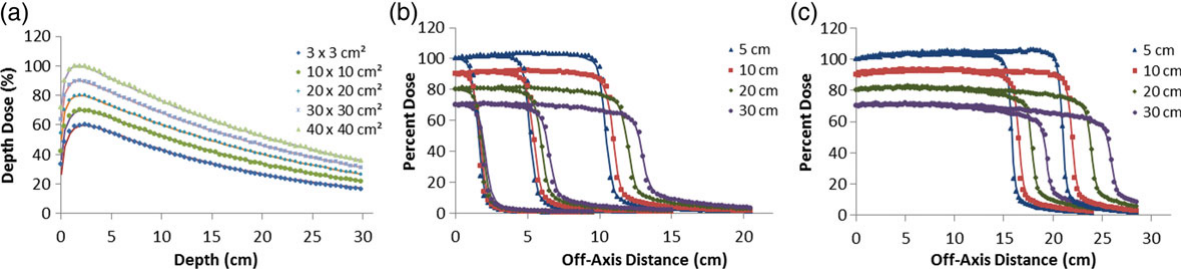
Comparison between MC (symbols) and GBD (lines): (**a**) PDD curves and (**b**), (**c**) dose profiles for 15-MV photon beam for 3 × 3 cm^2^, 10 × 10 cm^2^, 20 × 20 cm^2^, 30 × 30 cm^2^ and 40 × 40 cm^2^ fields. For clarity, the 30 × 30 cm^2^, 20 × 20 cm^2^, 10 × 10 cm^2^ and 3 × 3 cm^2^ fields are scaled by 0.9, 0.8, 0.7 and 0.6 respectively for PDD curves. Beam profiles are calculated at the depths of 5, 10, 20 and 30 cm. Curves for the depths of 10, 20 and 30 cm are scaled by 0.9, 0.8 and 0.7, respectively, for inclusion on the same graph. All curves are normalized to dose at d_max_ of central axis. Calculations are based on optimal parameters.

The 15-MV PDD curves for all field sizes are shown in Fig. 3a. Figure 3b and c show the 15-MV dose profiles for the 3 × 3 cm^2^, 10 × 10 cm^2^ and 20 × 20 cm^2^ and for the 30 × 30 cm^2^ and 40 × 40 cm^2^ fields, respectively. Excellent agreement between MC and GBD for PDDs and dose profiles was attained for optimal primary electron beam parameters. For all doses below d_max_, the average local difference between the MC and GBD PDD curves was within 0.4%. For dose profiles, the average local difference between the MC and GBD in the plateau region was within 1.1%. The 1.5-mm DTA limit was also fulfilled for the penumbra region. The MC and GBD S_cp_s agreed to within 0.9% (Table 1).

### Reproducibility evaluation of standard LINAC model

Tables 2 and 3 show the results of reproducibility evaluation of the standard LINAC model for 6- and 10-MV PDDs and for beam profiles with different field sizes. The mean and standard deviations of average local differences were calculated for beam data of different institutions. The excellent reproducibility for PDD curves for all field sizes and energies is evident. For the worst cases for 6- and 10-MV beams, the average local differences were 1.1% and 1.3%, respectively. Beam profiles showed excellent reproducibility for field sizes 3 × 3 cm^2^, 10 × 10 cm^2^ and 20 × 20 cm^2^. In the worst cases for 6- and 10-MV beams, the average local differences were 0.8% and 1.4%, respectively.

**Table 2.** Reproducibility evaluation of MC LINAC model for 6-MV PDDs and beam profiles: mean, standard deviation and range of average local differences of four sets of institutions' beam data are shown

Field Size (cm)	PDD		DOSE PROFILES							
			Depth = 50 mm		Depth = 100 mm		Depth = 200 mm		Depth = 300 mm	
	Mean ± SD (%)	(min–max)	Mean ± SD (%)	(min–max)	Mean ± SD (%)	(min–max)	Mean ± SD (%)	(min–max)	Mean ± SD (%)	(min–max)
										
3 × 3	0.6 ± 0.4	(0.3–1.1)	0.3 ± 0.0	(0.3–0.3)	0.3 ± 0.1	(0.3–0.4)	0.5 ± 0.2	(0.3–0.7)	0.5 ± 0.2	(0.3–0.8)
10 × 10	0.2 ± 0.2	(0.1–0.5)	0.6 ± 0.1	(0.5–0.6)	0.4 ± 0.0	(0.4–0.5)	0.4 ± 0.1	(0.3–0.4)	0.4 ± 0.1	(0.3–0.4)
20 × 20	0.3 ± 0.1	(0.2–0.5)	0.6 ± 0.1	(0.4–0.7)	0.5 ± 0.1	(0.4–0.6)	0.5 ± 0.1	(0.4–0.5)	0.7 ± 0.1	(0.6–0.8)
30 × 30	0.4 ± 0.1	(0.4–0.6)	0.9 ± 0.1	(0.8–1.0)	0.8 ± 0.1	(0.7–0.9)	1.0 ± 0.2	(0.7–1.2)	1.8 ± 0.6	(1.0–2.5)
40 × 40	0.5 ± 0.0	(0.5–0.6)	0.8 ± 0.1	(0.6–0.9)	0.9 ± 0.3	(0.7–1.3)	1.2 ± 0.2	(1.0–1.5)	1.6 ± 0.3	(1.3–2.0)

**Table 3.** Reproducibility evaluation of MC LINAC model for 10-MV PDDs and beam profiles: mean, standard deviation and range of average local differences of seven sets of institutions' beam data are shown

Field Size (cm)	PDD		DOSE PROFILES							
			Depth = 50 mm		Depth = 100 mm		Depth = 200 mm		Depth = 300 mm	
	Mean ± SD (%)	(min–max)	Mean ± SD (%)	(min–max)	Mean ± SD (%)	(min–max)	Mean ± SD (%)	(min–max)	Mean ± SD (%)	(min–max)
3 × 3	0.3 ± 0.1	(0.1–0.4)	0.4 ± 0.1	(0.2–0.5)	0.3 ± 0.2	(0.1–0.6)	0.5 ± 0.3	(0.2–1.2)	0.5 ± 0.4	(0.2–1.4)
10 × 10	0.2 ± 0.1	(0.2–0.4)	0.4 ± 0.1	(0.3–0.5)	0.5 ± 0.1	(0.4–0.6)	0.5 ± 0.1	(0.4–0.6)	0.7 ± 0.1	(0.6–0.9)
20 × 20	0.3 ± 0.1	(0.3–0.5)	0.4 ± 0.1	(0.3–0.5)	0.4 ± 0.1	(0.3–0.6)	0.5 ± 0.1	(0.4–0.7)	0.7 ± 0.1	(0.5–0.9)
30 × 30	0.4 ± 0.1	(0.3–0.6)	0.7 ± 0.1	(0.5–0.9)	0.9 ± 0.2	(0.7–1.2)	1.4 ± 0.2	(1.1–1.7)	1.2 ± 0.4	(0.8–1.7)
40 × 40	0.5 ± 0.3	(0.4–1.3)	1.1 ± 0.2	(0.9–1.4)	1.2 ± 0.3	(0.9–1.5)	1.1 ± 0.3	(0.8–1.5)	1.6 ± 0.5	(1.0–2.1)

Good reproducibility can be seen as well for large field sizes (30 × 30 cm^2^ and 40 × 40 cm^2^). For the worst cases for 6- and 10-MV, the average local differences were 2.5% and 2.1%, respectively.

**Fig. 4.**
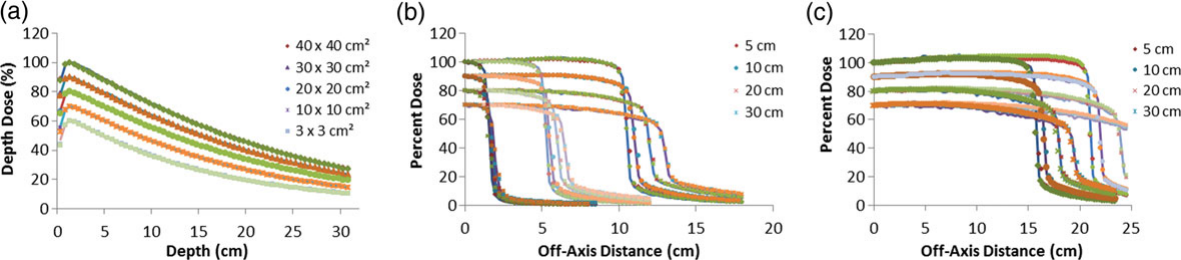
Best and worst match between MC (lines) and institutions' (symbols) data: (**a**) PDD curves and (**b**), (**c**) dose profiles for 6-MV photon beam for 3 × 3 cm^2^, 10 × 10 cm^2^, 20 × 20 cm^2^, 30 × 30 cm^2^ and 40 × 40 cm^2^ fields. For clarity, the 30 × 30 cm^2^, 20 × 20 cm^2^, 10 × 10 cm^2^ and 3 × 3 cm^2^ fields are scaled by 0.9, 0.8, 0.7 and 0.6, respectively, for PDD curves. Beam profiles are calculated at the depths of 5, 10, 20 and 30 cm. Curves for the depths of 10, 20 and 30 cm are scaled by 0.9, 0.8 and 0.7, respectively, for inclusion on the same graph. An excellent reproduction can be observed.

**Fig. 5.**
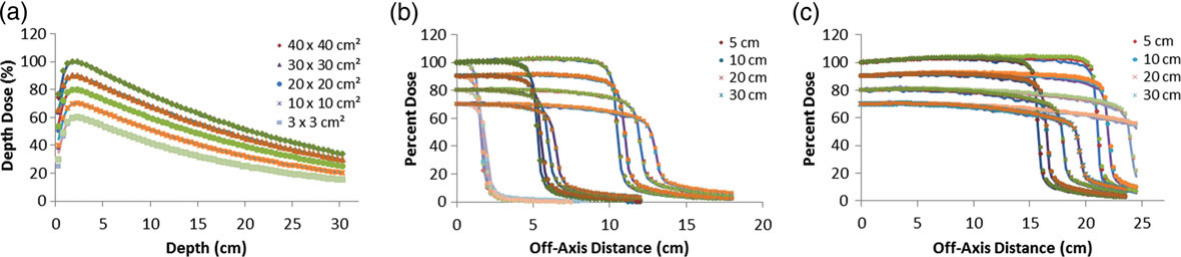
Best and worst match between MC (lines) and institutions' (symbols) data: (**a**) PDD curves and (**b**), (**c**) dose profiles for 10-MV photon beam for 3 × 3 cm^2^, 10 × 10 cm^2^, 20 × 20 cm^2^, 30 × 30 cm^2^ and 40 × 40 cm^2^ fields. For clarity, the 30 × 30 cm^2^, 20 × 20 cm^2^, 10 × 10 cm^2^ and 3 × 3 cm^2^ fields are scaled by 0.9, 0.8, 0.7 and 0.6, respectively, for PDD curves. Beam profiles are calculated at the depths of 5, 10, 20 and 30 cm. Curves for the depths of 10, 20 and 30 cm are scaled by 0.9, 0.8 and 0.7, respectively, for inclusion on the same graph. An excellent reproduction can be observed.

**Fig. 6.**
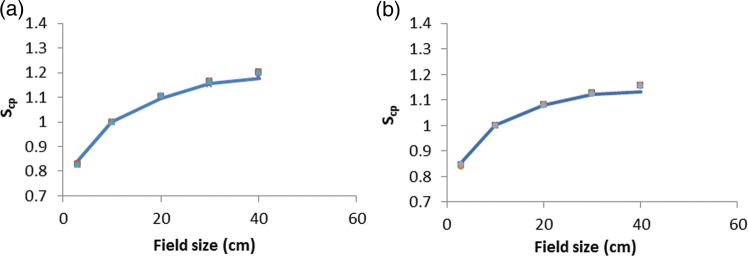
Comparison between MC (line) and institutions' (symbols) S_cp_ for (**a**) 6- and (**b**) 10-MV photon beams.

Figures 4 and 5 show a point-to-point best and worst match between MC and institutions' PDDs and beam profiles for 6- and 10-MV photon beams for all field sizes and depths. Excellent reproducibility can be seen here, too. Figure 6 shows a point-to-point comparison between MC and institutions' S_cp_s for 6- and 10-MV photon beams, while Table 4 presents a statistical comparison between MC and institutions' mean S_cp_s with standard deviations for all field sizes. For the worst cases for 6- and 10-MV beams, the observed differences were 1.6% and 2.0%, respectively.

**Table 4.** Comparison between MC and institutions' mean S_cp_s: ranges of institutions' S_cp_ and %Diff are shown for all field sizes

Field size (cm)	MC S_cp_	Institutions' S_cp_ Mean ± SD	(min–max)	% Diff	(min–max)
**6 MV**					
3 × 3	0.840	0.826 ± 0.002	(0.825–0.829)	1.6	(−2.2–1.2)
10 × 10	1	1			
20 × 20	1.094	1.105 ± 0.002	(1.103–1.107)	−1.0	(0.6–1.4)
30 × 30	1.157	1.160 ± 0.007	(1.150–1.165)	−0.3	(−1.0–1.5)
40 × 40	1.177	1.195 ± 0.008	(1.183–1.201)	−1.5	(0.2–3)
**10 MV**					
3 × 3	0.857	0.847 ± 0.004	(0.839–0.849)	0.4	(−0.4–1.1)
10 × 10	1	1			
20 × 20	1.081	1.081 ± 0.001	(1.079–1.083)	0.0	(−0.2–0.2)
30 × 30	1.122	1.126 ± 0.002	(1.124–1.130)	−0.4	(0.0–0.7)
40 × 40	1.133	1.156 ± 0.003	(1.153–1.161)	−2.0	(1.5–2.6)

## DISCUSSION

We developed an MC-based standard Varian LINAC model for independent verification of dose distributions submitted by participating institutions for QA of multi-institutional clinical trials and routine clinical practice. Many research studies have demonstrated that the MC method yields accurate calculations for dose distributions for clinical RT, particularly in heterogeneous tissues [13, 24–25].

Many investigators have used a variety of MC codes to develop MC models of photon beams from Varian linear accelerators for different objectives [26–38]. The accuracy of MC for PDDs, beam profiles and S_cp_s has been reported for a wide range of energies (4–18 MV). Table 5 summarizes the reported accuracy of these studies with related information about LINAC models, MC codes and beam energies. For PDDs and beam profiles, the reported accuracy is generally 2.0%, and within 1.5% for S_cp_s_._ In most cases, MC dose distributions were matched to measured dose distributions specific for each institution. In one case, Bednarz *et al*. tuned MC dose distributions to match GBD.

**Table 5.** Comparison of this work with other studies that used MC codes to model photon beams from Varian LINACs

Author	Accelerator	MC code	Energy (MV)	Reported accuracy (%)		
				PDD	Profiles	Scp
Chibani *et al*. [26]	21EX, 2300C/D	GEPTS	4, 6, 10, 15, 18	1	1	1.2
Bednarz *et al*. [27]	2100C	MCNPX	6, 18	2	2	
Yamamoto *et al*. [28]	2300C/D	EGS4*/*PRESTA	15	1	1.5	
Mesbahi *et al*. [29]	2100EX	MCNP4C	6, 15	1.5	2	
Popescu *et al*. [30]	21EX	BEAMnrc	6			1.3
Cho *et al*. [31]	2100	BEAMnrc	6	1.5	2	1
Ding [32]	2100EX	BEAMnrc	6, 18			1.5
Siantar *et al*. [33]	2100C	PEREGRINE	6, 18	2	2	1.3
Fix *et al*. [34]	2300C/D	GEANT	6	1.5	1.5	1.5
Ma *et al*. [35]	1800, 2100C, 2300CD	EGS4/BEAM	4, 6, 15	2	2	
Deng *et al*. [36]	2100C, 2300CD	EGS4/BEAM	4, 6, 15	2	2	
Liu *et al*. [37]	2100C	EGS4/BEAM	6, 10, 18	2	2	
Lovelock *et al*. [38]	600C, 2100C	EGS4	6, 15	1	3	
This study	23EX	BEAMnrc	6, 10, 15	1	1.5	1.2

In this study, 6-, 10- and 15-MV photon beams were characterized by using the BEAMnrc code. Our results were generally consistent with and more accurate than the beam data reported by other authors. For all energies and field sizes, accuracies were attained to within 1.0% for PDDs, 1.5% for beam profiles, and 1.2% for S_cp_s.

Our standard LINAC model has been developed for a wide range of field sizes from 3 × 3 cm^2^ to 40 × 40 cm^2^. Based on the accurate modeling of the accelerator head geometry, MC simulations have been proven to match GBD very exactly. Overall, our results are in excellent agreement with GBD, and this agreement can be attributed to the rigorous tuning procedure that was used to determine the optimal initial electron beam parameters for the accelerator model.

To evaluate the reproducibility of the standard LINAC model developed by us, MC-based simulations and institutions' PDDs and beam profiles were compared for 6- and 10-MV beams, while mean and standard deviations of average local differences were calculated for statistical analysis. For both energies, the mean and standard deviations were within 1.0% for PDDs and within 2.0% for beam profiles at all depths. For S_cp_s, the MC and institutions' mean values agreed to within 2.0%. We used the 95% confidence level for estimating the probability range, and the 95% confidence intervals for the worst cases for 6-MV were 1.4% for PDDs, 3.0% for beam profiles and 2.2% for S_cp_s_._For 10 MV the corresponding worst values were 1.1%, 2.6% and 2.6% for PDDs, beam profiles and S_cp_s, respectively.

Although 4, 6 and 10 MV are widely used beam energies for treatment in Japan, the evaluation of the LINAC model was performed for 6- and 10-MV dose distributions only because data for 4-MV beams was not included in GBD provided by the vendor. For a future evaluation study, however, we plan to use mean values of 4-MV beam data collected from a large number of institutions. In addition, despite the fact that 15 MV is currently not included in the beam datasets provided by institutions, our developed model can be evaluated subsequently and employed for future clinical trials utilizing 15-MV photon beams.

Modeling the Varian LINACs was selected as the first priority for our study. This was due to the fact that (to the best of our knowledge) the share of Varian LINACs is larger than those of other vendors, especially in advanced academic and clinical institutions, and standard beam data for a variety of energies were available. To facilitate more comprehensive multi-institutional clinical trials, our LINAC model could be extended by accounting for beam modifiers and simulating the treatment heads based on the specifications of other vendors.

The standard LINAC model developed by us and evaluated in this study was commissioned with GBD, because it is very time consuming and impractical to commission it with beam data provided by individual institutions. On the other hand, it may be possible to develop a LINAC model for individual institutions with beam data provided by a given institution, although this would be very time consuming. Nevertheless, if this becomes possible, we will be able to obtain more accurate dose distributions, and this may enable us to estimate a more accurate dose–response relationship in combination with clinical data.

We are developing an informatics infrastructure similar to that of the image-guided therapy center to support the submission, archiving, retrieval and QA review of advanced technology protocol data for patients registered for multi-institutional clinical trials [39]. In conjunction with this infrastructure, our LINAC model can be used as an MC-based independent dose-distribution verification system for QA of multi-institutional clinical trials and routine clinical practice in Japan.

## CONCLUSIONS

An MC-based standard Varian LINAC model was developed as a preliminary requirement to independently verify dose distributions submitted by participating institutions for QA of multi-institutional clinical trials and routine clinical practice. Based on the accurate modeling of the accelerator head geometry, MC dose distributions were tuned to match GBD very accurately. Reproducibility evaluation further substantiated the capability of our model.

This work was motivated by the need to develop an independent dose verification system for QA of multi-institutional clinical trials and routine clinical practice. Our standard LINAC model can be expected to function as a pivotal tool for ensuring that participating institutions deliver prescribed doses that are consistent with the requirements of clinical protocols, and for validating the outcomes of clinical trials.

## FUNDING

This work was supported by a JSPS Core-to-Core program from the Japan Society for the Promotion of Science (Grant No. 23003).
